# Simulation-based training in minimally invasive surgical therapies (MIST): current evidence and future directions for artificial intelligence integration—a systematic review by EAU endourology

**DOI:** 10.1007/s00345-025-05834-8

**Published:** 2025-07-18

**Authors:** Carlotta Nedbal, Vineet Gauhar, Thomas Herrmann, Abhishek Singh, Ali Talyshinskii, Feras Al Jaafari, Bhaskar Kumar Somani

**Affiliations:** 1 Polytechnic University Le Marche, Ancona, Italy; 2 Urology, ASST Fatebenefratelli Sacco, Milan, Italy; 3 Endourology Section, European Association of Urology, Arnhem, The Netherlands; 4 Urology, Ng Teng Fong General Hospital, Singapore, Singapore; 5 Asian Institute of nephrourology (AINU), Nungambakkam, Chennai, India; 6 Kantonspital FrauenfeldSpital Thurgau AG, Frauenfeld, Switzerland; 7 Department of Community Medicine and Public Health, King George’s Medical University, Lucknow, Uttar Pradesh India; 8 Astana Medical University, Astana, Astana Kazakhstan; 9 School of Medicine, University of St Andrews and Victoria Hospital, NHS Fife, Kirkcaldy, Scotland, UK; 10 University Hospital Southampton NHS Foundation Trust, Southampton, UK

**Keywords:** Simulation, MIST, BPH, Artificial intelligence, Training

## Abstract

**Introduction:**

Benign prostatic hyperplasia (BPH) affects a growing proportion of the aging male population. Minimally invasive surgical therapies (MISTs) such as Rezum and UroLift offer effective alternatives to traditional approaches like transurethral resection of the prostate (TURP). However, training in these procedures is challenged by limited case exposure and variability across residency programs. Simulation-based training has emerged as a valuable tool to enhance surgical education. This study aims to assess the current evidence on simulation-based training for Rezum and UroLift, evaluating its validity, effectiveness, and potential integration with artificial intelligence (AI) in urology education.

**Materials and methods:**

A systematic literature review was conducted on March 11, 2025, across PubMed, Scopus, Cochrane, and Google Scholar following PRISMA guidelines. Search terms included combinations of MIST techniques (Rezum, UroLift, iTIND) and training modalities (simulation, virtual reality, artificial intelligence). Studies were selected using PICOS criteria, focusing on urology trainees undergoing simulation-based training. Preclinical, review, and non-English studies were excluded.

**Results:**

only 3 studies met the inclusion criteria: one focused on Ron between junior and senior residents, especially in implant placement and procedural technique. Simulation was highly rated by trainees in workshop settings, though predictive validity remains unproven.

**Conclusion:**

Simulation-based training for Rezum and UroLift is a promising method to enhance resident competency in MIST procedures. Current evidence supports its face, content, and construct validity, though further studies are needed to confirm predictive validity and optimize training protocols. Integration of AI and telementoring may further improve training effectiveness and accessibility across institutions.

## Introduction

The increasing prevalence of benign prostatic hyperplasia (BPH) among the aging population has led to the development of minimally invasive surgical therapies (MISTs) like Rezum and UroLift [1, 2]. These are promising alternatives to transurethral resection of the prostate (TURP), which has been the gold standard for BPH management [3]. MISTs compare favourably to holmium laser enucleation of the prostate (HoLEP) in terms of tolerability and patient satisfaction, particularly due to their ejaculation-sparing capabilities [4]. However, mastering these techniques is challenging due to limited case volumes, time constraints, and variations in resident exposure [5]. As surgical technologies advance, effective training solutions are urgently needed to bridge the gap between theoretical knowledge and practical application.

Simulation-based training has emerged as a crucial component of modern urology residency programs [6]. By providing a controlled, risk-free environment, surgical simulators allow residents to practice and refine their skills in techniques such as Rezum and UroLift, helping to shorten the learning curve and ultimately improve both technical competence and patient safety [7]. Innovations in simulation technology, including virtual reality (VR) platforms, high-fidelity mannequins, and hybrid task trainers, have revolutionized surgical education by offering residents experiences that closely mirror actual clinical practice [8, 9]. These tools allow for repetitive practice and the development of muscle memory, which is essential for mastering complex procedures.

Simulation training has proven to be effective not only for skill acquisition but also as a valuable tool for performance evaluation, providing both formative and summative assessments. Despite their growing use, there remains limited research on the construct validity of some simulation platforms, such as those for Rezum and UroLift. As urology training programs increasingly adopt simulation to supplement traditional methods, evaluating the effectiveness and validity of these platforms becomes crucial to ensuring they provide meaningful educational experiences. This article explores the role of artificial intelligence (AI) and simulation in MIST training, specifically focusing on the potential of Rezum and UroLift simulators. It examines how these tools are shaping the future of urology education, improving training efficiency, and enhancing overall patient outcomes.

## Materials and methods

### Literature search

A systematic review of the current literature was performed on 11th March 2025, searching Medline (PubMed), Scopus, Cochrane and Google Scholar for eligible studies. The search strategy was conducted following the Preferred Reporting Items for Systematic Reviews and Meta Analyses (PRISMA) statement [10]. The search strategy included both Medical Subject Headings (MeSH) and free-text terms, using the following keywords: (“rezum” OR “iTIND” OR “Urethral lift” OR “UroLift”) AND (“artificial intelligence” OR “machine learning” OR “artificial neural network” OR “automatic prediction” OR “big data” OR “deep learning” OR “simulation” OR “phantom” OR “3D model” or “virtual reality” OR “augmented reality” OR “training”).

### Screening and selection

**Fig. 1:**
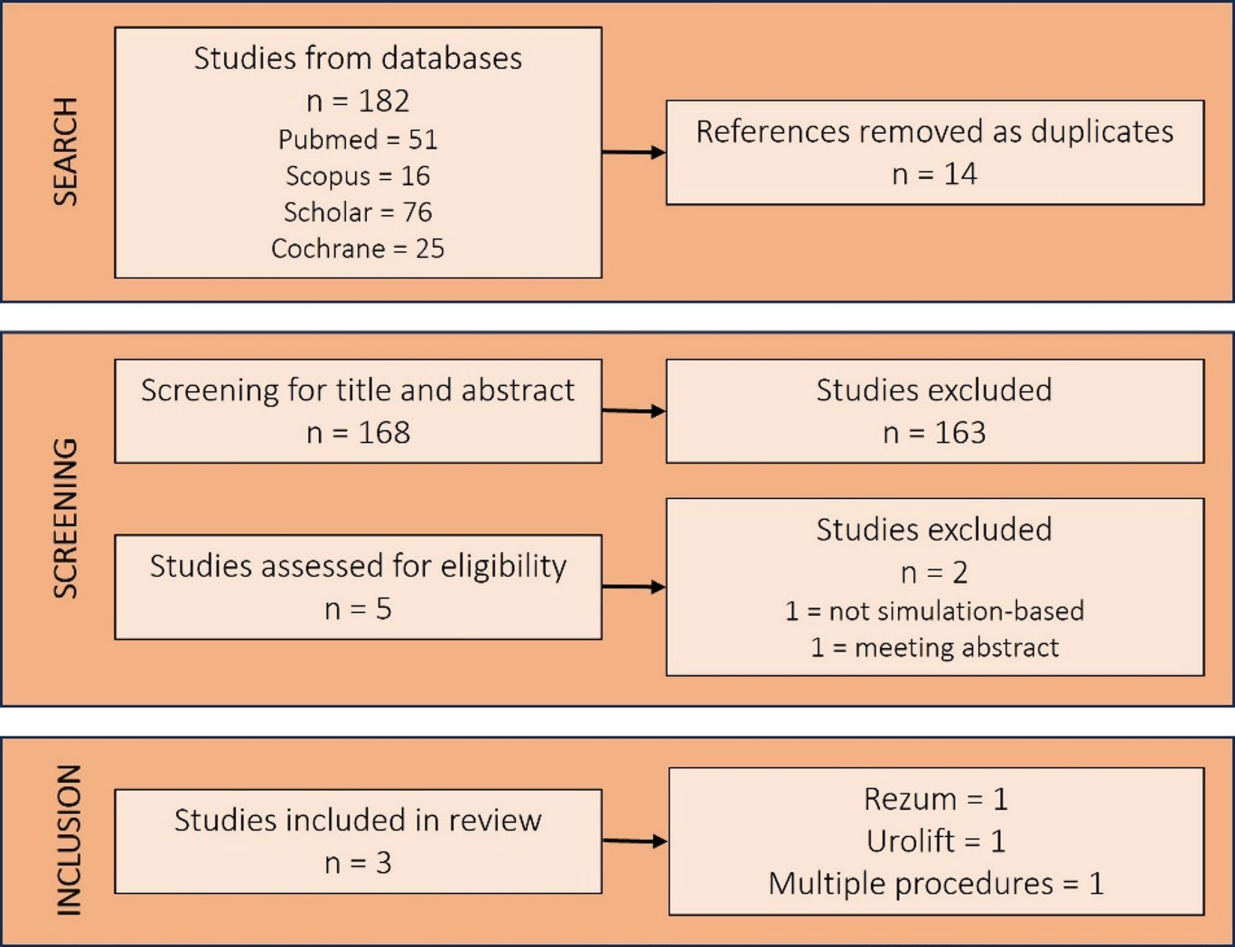
Flow diagram of the literature search, screening, and study selection process

The PICOS (Patient, Intervention, Comparison, Outcome, Study type) model was used to frame and answer the clinical question: P: trainees (medical students, residents, young urologists, urologist in specific training); I: Rezum, UroLift or iTIND simulation; C: none or any other simulation, simulation of other BPH procedures; O: safety, efficacy, learning curve; S: comparative, retrospective/prospective, non-randomised/randomised studies. Two independent authors (CN, VG) screened the retrieved studies according to the PICOS eligibility criteria, accepting only English language articles. Discrepancies were resolved by a third senior author (BKS). At screening, preclinical/laboratory studies, case reports, reviews, letters to the editor, and meeting abstracts were excluded. For papers meeting the requirements, full text was retrieved and further screened. Figure 1 shows details of the selection process.

## Results

Literature search retrieved 3 papers. No study investigating simulation-based training for iTIND was retrieved, therefore this procedure will not be analyzed in the results. Among the included papers, one study discussed simulation for UroLift, one discussed simulation for Rezum, and the third disclosed the role of simulation for different BPH procedures, including both UroLift and Rezum (Table 1).

When evaluating simulators for MIST in the treatment of BPH, it is essential to consider their face, content, and construct validity:

- Face Validity refers to how realistic and credible the simulator appears to expert users. It assesses whether the simulation looks and feels like the real procedure, making it intuitively acceptable for training purposes.
- Content Validity examines whether the simulator includes all the essential components and steps necessary for performing MIST procedures. This ensures comprehensive coverage of the skills and knowledge required.
- Construct Validity evaluates whether the simulator can differentiate between users of varying skill levels. A simulator with high construct validity can distinguish novices from experts based on performance metrics.

### Rezum simulator

The only available manuscript investigating the role of Rezum simulator in urology training was published in 2024 by Alsowayan and colleagues [11]. Participants, divided into four levels of expertise (consultants, senior registrars, senior residents, and junior residents), performed 3 simulated procedures per person (51 in total) on a VR simulator. In this study, the authors used the Rezum simulator, created by Boston Scientific, which utilizes the VirtaMed platform and offers a range of training scenarios in a 3D virtual environment. Multiple performance metrics can be assessed, such as the number of successful treatments, presence of contagious lesions, procedure duration, total saline used, partial treatments, vapor leakage, procedures conducted with limited visibility, and excessive torque applied to the lobe. The software assigns scores to each metric based on specific predefined objectives and generates an overall performance score by integrating all these factors.

Despite the absence of a punctual validation, the simulator was found consistent and useful by Alsowayan, who reported high reliability values after the training procedures. The authors also found better performance scores among the senior participants (consultants, senior registrars), but no difference in procedural time (Table 2).

### UroLift simulator

The other two studies included in our review evaluated UroLift simulation on the same platform (Table 2). The UroLift simulator developed by NeoTract is an interactive, web-based 3D training system powered by the VirtaMed platform. It monitors performance through three key report categories: device operation, procedural technique, and implant positioning. The simulation evaluates a range of skills, including deployment (total and successful deployments, lateral suture centering), precision (proximal centering, mucosal and proximal mucosal abrasion), and implant placement (implants positioned in proximal and distal zones, as well as those placed too close to the bladder).

Chow and colleagues [12] evaluated urology residents’ experiences with surgical simulation training across multiple institutions. Residents participated in hands-on workshops covering various procedures, including UroLift. Most participants (84%) rated the training highly, with 97% finding it beneficial and 67% believing it should be required in residency. The results highlight the value of simulation as an effective training tool in urology education. However, this study did not perform a direct evaluation and validation of the simulator.

A subsequent study by Alzahrani and colleagues [13] assessed the construct validity of UroLift simulation training by comparing the performance of junior and senior urology residents. Participants completed three cases of varying difficulty, with performance analysed using statistical tests. Significant differences were found between the groups in skills such as proximal centering, mucosal abrasion, and implant placement in proximal zones, while other metrics showed no significant variation. The findings suggest that UroLift simulations are valuable for training, though further refinement may be needed for objective performance evaluation. Interestingly, in this study simulators offered difficulty levels with a prostate volume up to 90 g, by far larger than what is recommended in the EAU guidelines.

**Table 1:** Characteristics and details of the included studies

Study	Country	Date of study	Type of study	Procedure(s)	No. of trainees	Trainees’ level of expertise	No. of procedures
Alsowayan, 2024 [11]	Saudi Arabia	April 2021	Prospective	Rezum (Boston Scientific)	17	consultants (4), senior registrars (4), senior residents (4), and junior residents (5).	51 (3 each)
Alzahrani, 2022 [13]	Saudi Arabia	N/A	Prospective	UroLift (NeoTract, Pleasanton, CA)	8	senior residents (*N* = 4), junior residents (*N* = 4)	24 (3 levels each)
Chow, 2017 [12]	USA	2014–2017	Retrospective	UroLift (NeoTract, Pleasanton, CA) Miscellaneous (robotic, laparoscopy, URS, Green light PVP, botox injection)	75	Urology residents: PGY-1 (11), PGY-2 (16), PGY-3 (9), PGY-4 (15), PGY-5 (6), and PGY-6 (2).	N/A

**Table 2:** Results on simulators for training from the three included studies

Study	Type of simulation	Details	Reliability	Efficacy	Conclusion
Alsowayan, 2024 [11]	3D virtual simulator (VirtaMed platform)	Varied metrics (lesion, procedure time, cumulative saline use, incomplete treatments, vapor leakage, poor visualization, excessive torque)	Good reliability (ICC reliability tesy)	Significant variance in the total scores among candidates. No statistically significant result in procedure time between the candidates.	The Rezum simulation is a valid, reliable simulator of most of its metrics.
Alzahrani, 2022 [13]	3D virtual simulator (VirtaMed platform)	Varied metrics (Deployment skill, Precision skill, Implants skil) for 3 levels of difficulty (easy, medium, difficult)	Not assessed	Significant differences among junior residents and senior residents in precision and implant skills. No difference in deployments.	UroLift simulations are useful for training as a practicing tool. All trainees could practice this novel prostatic surgery in a safe environment; their performance improved across all cases and trials.
Chow, 2017 [12]	3D virtual simulator (VirtaMed platform)	Scoring calculated by the simulation device, based on surgical technique, efficiency of movement, and efficacy of treatment as calculated by the simulation device.	Excellent face validity	Highly scored (9 on 1–10 score)	Surgical simulation is beneficial and should be a requirement for Urology residency. High ratings of usefulness for each exercise demonstrated excellent face validity.

## Discussion

The integration of surgical simulation into urology residency training has demonstrated significant benefits for all levels of expertise. Simulation provides a controlled, risk-free environment for trainees to develop technical proficiency, overcome learning curves, and enhance patient safety [14]. The increasing attention towards controlled training with simulators highlights the growing role of simulation in urological education, with a focus on multi-institutional collaboration, validity testing, and the impact of trainee involvement on procedural outcomes [15]. With several studies already focusing on simulators for well-known and standardised procedures (i.e., prostatic enucleation [16], ureteroscopy [17], robotic procedures, etc.), there is still little evidence on the role of simulators in the education toward MISTs. As these mini-invasive treatment options are spreading, we expect further studies focusing on the education and the real-time application with AI-powered tools and simulators.

Validation studies of the Rezum and UroLift simulators are limited but promising. Alsowayan [11] and colleagues demonstrated construct validity for the Rezum simulator, with senior residents and consultants outperforming junior trainees in metrics such as contiguous lesion formation and total score. Similarly, Alzahrani [13] and Alsowayan [11] found that senior residents performed better in UroLift simulations, particularly in proximal centering and mucosal abrasion. However, not all metrics showed significant differences, suggesting the need for further refinement of these simulators. High reliability scores indicate consistent performance measurement, supporting their use in structured training programs.

Alam and colleagues [18] examined the involvement of residents in UroLift procedures and found no adverse effects on patient outcomes, although junior residents required longer operative times. This finding is in line with the broader surgical literature, in which resident participation is associated with longer procedure duration but not with compromised outcomes. The study also observed that senior residents achieved operating times comparable to those of senior surgeons, underscoring the role of graded responsibility in training. Despite not evaluating young urologists’ performance in a simulate setting, these results suggest that simulation may bridge the initial learning curve of MIST procedures, preparing residents for real-world practice. In addition, they could have a profound impact on residents’ training, leading to early certification and achievement of autonomy.

The multi-institutional workshop model described by Chow [12] emphasizes the feasibility and effectiveness of shared resources in urology training. By pooling simulators and faculty expertise, this model solves financial and logistical barriers by offering residents exposure to a variety of procedures, including the UroLift. Participants rated the workshops highly in terms of usefulness, with 97% reporting educational benefits and 67% favouring simulation as a requirement for residency. This model could be adapted for training on Rezum and UroLift, providing broader access to these technologies.

Despite the benefits, gaps remain in the validation and integration of MIST simulators into residency programs. In 2023, Luu and Gonzalez [5] point to the lack of predictive validity studies for these technologies, which are critical for assessing long-term skill retention and clinical performance. They highlighted a significant shift from traditional surgical techniques like TURP to a diverse array of laser therapies and MISTs. Despite the growing number of FDA-approved procedures, training challenges persist due to the complexity and variability in access to new technologies [19]. Simulators, especially VR models, have emerged as valuable tools in overcoming learning barriers and enhancing surgical proficiency [20]. Various simulators for TURP, laser enucleation, and MISTs have been developed and validated to different extents [21], although some, like iTIND, lack published validation studies. The authors underscored that learning curves vary widely by procedure, with laser techniques generally requiring more extensive training. Structured training programs, simulation-based task deconstruction, and mentorship are key strategies identified to accelerate skill acquisition and ensure patient safety [22].

AI has been applied to manage various urological conditions, including BPH, by enhancing diagnostics, risk stratification, and patient care [23, 24]. However, its integration into training and clinical guidelines is limited. For AI to transform training, simulation, and routine BPH care, it must be developed, validated, and integrated into clinical pathways. AI-driven simulators are revolutionizing endourological training with adaptive, feedback-driven learning environments. Similar integration is necessary for both standard procedures (enucleation, TURP) and MIST used in BPH treatment, such as UroLift, Rezum, and iTIND, to standardize technique, accelerate proficiency, and improve patient outcomes [25]. Embedding AI in the education and simulation of procedures such as UroLift, Rezum, and iTIND could help standardize technique, accelerate proficiency, and improve patient outcomes.

The present study, while underscoring the current and possible future applications of AI and simulators for MISTs, is limited by the lack of thorough literature evidence. Our search only retrieved three eligible studies, thus depicting the lack of standardised evidence. Moreover, only two (UroLift and Rezum) of the available MISTs for BPH have been investigated through published research. This highlight the limited current evidence, that does not include comparative and more broad studies. Another limitation is in regards of AI applications, that are still mostly theoretical and not thorough applied in MIST simulators. Still, a discussion of future applications and developments is provided based on available evidence for both BPH and other urological field. Consequently, the present study was not able to perform a comprehensive evaluation of AI applications for MIST simulation, and important factors such as predictive validity remain unaddressed.

**Fig. 2:**
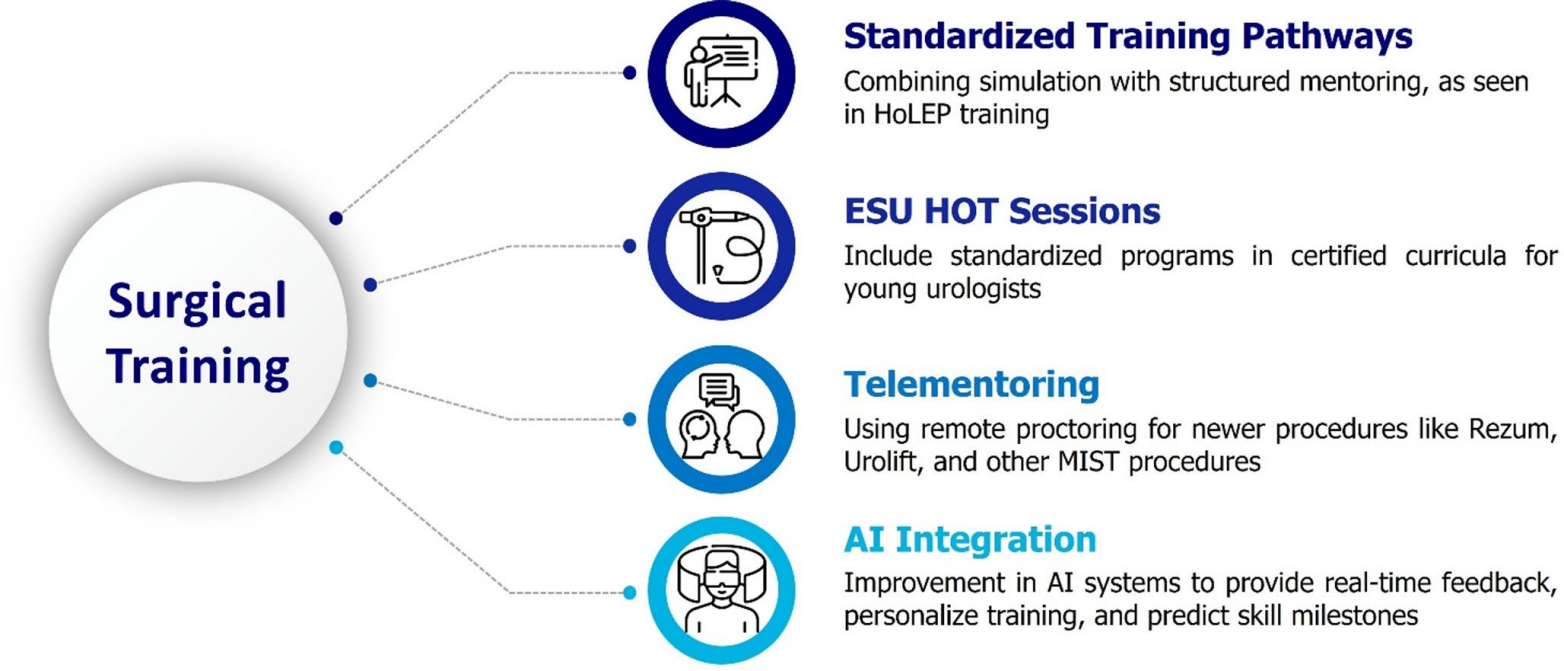
Area of improvement for future research

Future research (Fig. 2) should focus on creating standardized training pathways, combining simulation with structured mentoring, as seen in HoLEP training, to optimize skill acquisition. Such programs could be included in European School of Urology (ESU) practical training sessions and certified curricula, offering consistency and standardisation in surgical education to young urologists [23]. Another area of research could be telementoring [24]: this technology using remote proctoring, particularly for newer procedures such as aquablation, could be extended to Rezum, UroLift, and other MIST procedures to ensure standardized teaching programs and availability despite geographic barriers. In addition, MIST will likely see an improvement in AI integration in the near future, with new technologies incorporating AI systems to provide real-time feedback, personalize training, and predict skill milestones.

## Conclusion

Simulation-based training for Rezum and UroLift (and other MISTs) holds great promise for urology education, offering a safe and effective way to master these technologies. While current evidence supports their face, content, and construct validity, further studies are needed to establish predictive validity and optimize training protocols. By addressing these gaps and embracing innovative approaches like AI and telementoring, the urological community can ensure that residents are proficient in MIST procedures, ultimately improving patient care and outcomes.

## Data Availability

No datasets were generated or analysed during the current study.
